# Investigating the barriers to teaching family physicians' and specialists' collaboration in the training environment: a qualitative study

**DOI:** 10.1186/1472-6920-9-31

**Published:** 2009-06-07

**Authors:** Marie-Dominique Beaulieu, Louise Samson, Guy Rocher, Marc Rioux, Laurier Boucher, Claudio Del Grande

**Affiliations:** 1 Doctor Sadok Besrour Chair in Family Medicine, Department of Family Medicine, Universite de Montreal, Montreal, Canada; 2 Centre hospitalier de l'Universite de Montreal Research Centre (CRCHUM), Universite de Montreal, Montreal, Canada; 3 Department of Radiology, Radiation Oncology and Nuclear Medicine, Faculty of Medicine, Universite de Montreal, Montreal, Canada; 4 Department of Sociology and Centre de recherche en droit public (CRDP), Universite de Montreal, Montreal, Canada; 5 Le Protecteur du citoyen's Office, Government of Quebec, Montreal, Canada

## Abstract

**Background:**

Collaboration between physicians in different specialties is often taken for granted. However, poor interactions between family physicians and specialists contribute significantly to the observed discontinuity between primary and specialty care. The objective of this study was to explore how collaboration between family physicians and specialists was conceptualised as a competency and experienced in residency training curricula of four faculties of medicine in Canada.

**Methods:**

This is a multiple-case study based on Abbott's theory of professions. Programs targeted were family medicine, general psychiatry, radiology, and internal medicine. The content of the programs' objectives was analyzed. Associate deans of postgraduate studies, program directors, educators, and residents were interviewed individually or in focus groups (47 residents and 45 faculty members).

**Results:**

The training objectives related to family physicians-specialists collaboration were phrased in very general terms and lacked specificity. Obstacles to effective collaboration were aggregated under themes of professional responsibility and questioned expertise. Both trainees and trainers reported increasing distances between specialty and general medicine in three key fields of the professional system: the workplace arena, the training setting, and the production of academic knowledge.

**Conclusion:**

The challenges of developing collaborating skills between generalists and specialist physicians are comparable in many ways to those encountered in inter-professional collaboration and should be given more consideration than they currently receive if we want to improve coordination between primary and specialty care.

## Background

Interactions between family physicians (Appendix 1) and specialists significantly determine the quality of coordination between primary and specialty care. [1-3] Disruption of this coordination undermines healthcare system efficiency, quality of care, and patient safety.[2,4-6] Medical organizations worldwide have identified improving this collaboration as a priority.[3,7,8] Despite successful local interventions to improve such collaboration, results overall have been mitigated. [9-11]

It is during training that professional identity is shaped and professionals have their first collaboration experiences.[12,13] Empirical research exploring how professional collaboration is learned within the medical profession, particularly between family physicians and specialists, is relatively rare.[14] Attention has mainly been focused on teaching inter-professional collaboration, i.e., collaboration between health care professionals from different professions (nursing, social work, etc.).[15] Teaching collaboration between physicians has been mostly limited to the teaching of referral and consultation skills.[16,17]

The aim of this study was to develop a deeper understanding of how generalist-specialist collaboration is learned within medical schools, using Abbott's systemic theory of professions as the theoretical framework.[13] This framework integrates concepts of professional identity, professionalism, and professional interactions–all central to collaboration. According to Abbott's framework, the concept of specialization, and therefore of expertise, is fundamental to the professional system. Every profession must constantly defend its legitimacy and jurisdiction to preserve its social status among the system's other professions in three key fields: the *workplace arena*, where professional boundaries are negotiated on a day-to-day basis; the *training *field, where professional identity is shaped and commitment to the profession is inculcated; and the *academic knowledge *field, which legitimizes professional work and provides opportunities for developing new expertise.[12,13] Abbott also specifies that, in any profession, members' ability to differentiate themselves from each other is fundamental. This differentiation supports member autonomy and the pursuit of personal aspirations, but can also generate tension. According to this conceptual framework, the educational system, where future professionals are trained and academic knowledge evolves, is a key determinant of professional collaboration.[18]

The study was carried out in four Canadian medical faculties. We chose to target postgraduate training programs, where the professional identity specific to each medical discipline is shaped. Our research questions were the following: How is collaboration between family physicians and specialists approached in the formal curriculum of such programs? How is it experienced day-to-day in academic medical centers that constitute for trainers and trainees a workplace, a teaching setting, and a setting where knowledge is produced and transferred?

### Context of the study

**Figure 1:**
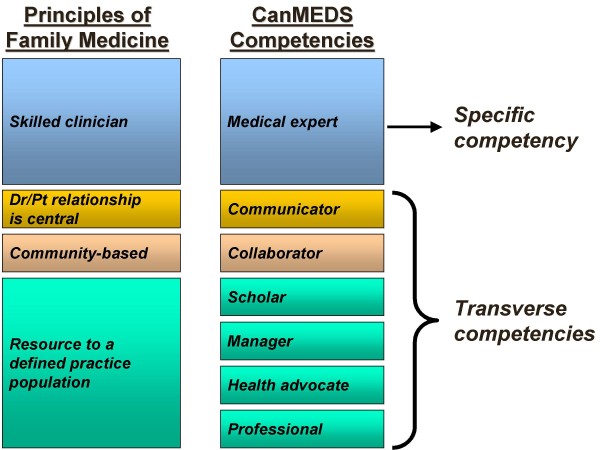
**Mapping of the four principles of family medicine and the seven CanMEDS competencies**.

While medical practice in Canada is under provincial jurisdiction, training programs are nationally accredited. Although each of the country's 17 medical schools has its individuality, core programs must respect national standards. Competencies are defined at the national level by the College of Family Physicians of Canada (CFPC) and the Royal College of Physicians and Surgeons of Canada (RCPSC). In 2000, the RCPSC revised its approach to training and program accreditation, adopting a competency-based approach. The foundational document, CanMEDS 2000[19], proposes seven competencies, including professionalism and collaboration. In contrast, the CFPC's approach to training and program accreditation is based on four principles considered to be the founding values of family medicine[20] (Figure 1). Postgraduate-level trainees are "residents"; family medicine residencies last two years and specialty programs, five. Family medicine residents are attached to a family medicine teaching practice throughout their residency, generally in the community setting.

## Methods

### Design and study population

This study used a multiple-case design[21], each case being a medical school. In 2003–2004, four Canadian medical schools were purposefully selected to contrast on two characteristics: geographical region (Eastern Canada, Quebec, Ontario, and Western Canada) and mission (orientation towards community and general practice (n = 2), or specialized care and research (n = 2)), since it was suspected that medical schools' missions may affect how collaboration was addressed in the formal curriculum. The researchers' school was excluded from the sample.

Residency programs targeted were family medicine and three specialty programs–general psychiatry, internal medicine, and radiology–that interact often with general practice. The choice of the latter may surprise, but radiologists are trained, among other things, to act as consultants to general practitioners on the appropriate choice of imaging technologies to investigate patients, and in community settings and non academic hospital centres, they do assume this role. In each case, four categories of respondents were enlisted to reconstitute the training continuum: the associate deans of postgraduate studies and each program's director, educators, and final-year residents. Individual and group interviews were conducted with these key informants.

### Participant selection and interview process

In selecting respondents, we first enlisted the associate deans' support. Then, the program directors were invited to participate. Once these persons had agreed to participate, a site visit was organized. Residents and educators were invited through the program directors' offices, respecting the Ethics Board's privacy guidelines. For each case, our target was four educators per program, five or six family medicine residents, and up to four residents in each specialty, with the aim of ensuring representation of the variety of the programs' teaching settings. Respondents had to be available when the research team was on site. Associate deans and program directors were interviewed individually. Specialty program educators and residents were met either in groups or individually, to accommodate schedules and numbers of participants (most specialty programs had fewer than four final-year residents). All family medicine residents were interviewed in focus groups. These two interview methods were not employed for triangulation, but for logistic reasons. Focus group methodology has inherent limitations: greater superficiality due to the number of participants, and the influence of group dynamics, or "group censoring".[22] To counter these phenomena, we frequently sought input from around the table and served as devil's advocate.

Our interview guide covered respondents' visions of family physicians' and specialists' roles; their experiences of generalist-specialist collaboration; and their teaching experiences and perceptions of the importance attributed to this competency. The interview script was comparable for all categories of respondents, with minor adaptations to refer to their specific position (i.e. resident, educator, associate dean) when appropriate. After a general introduction presenting the study's objectives in the context of the evolving roles of physicians within the health care system, we began with the same question for all the respondents: "How do you see the role of specialists and family physicians in the Canadian health care system?" We then asked the respondents how the training prepared them, or their trainees, to play their respective roles and to collaborate with one another. We asked about their day-to-day experiences of collaboration, and the barriers and facilitators they encountered. We concluded with a broad question on their perceptions of the current challenges in medical education.

Interviews were conducted by the researchers (MDB, LB; LS). Group interviews lasted 90 minutes and individual interviews, 45–60. Interviews were transcribed for analysis. The project was approved by the Research Ethics Committee of the Centre hospitalier de l'Universite de Montreal (CHUM) Research Centre.

### Analyses

The immersion/crystallization method[23] was used to analyze the data. Each researcher read all interview transcripts and consulting notes. Bringing their respective disciplines (sociology, social work, family medicine, specialized medicine) to the analysis, they crystallized out the most significant elements. They then combined their interpretations and compared themes and sub-themes; differences were resolved through consensus. Word processing software was used to code transcripts by themes.

Two investigators (MDB, LS) independently reviewed the programs' official documents and extracted information pertaining to generalist-specialist collaboration. The analysis studied stated objectives, their type (institution-specific or from CanMEDS 2000[19]) and degree of specificity, i.e., from general (e.g. collaborate with family physicians) to specific (e.g. explain the treatment plan).

## Results

In all, 27 individual and 14 group interviews were conducted with 92 respondents, providing a comprehensive sample. All associate deans and all program directors, with the exception of two in radiology, participated. Table 1 presents our respondents' characteristics. Because no salient mission-based differences were observed between schools, this parameter was discarded during analysis. Results are organized in two sections: generalist-specialist collaboration in the formal curriculum; and collaboration as experienced in practice in academic medical settings.

**Table 1:** Description of respondents

		**Gender**		
			
**Respondent category**	**Mean age (years)**	**Male** N	**Female** N	**TOTAL**
***Residents***				
Family medicine	26.3	12	17	29
Specialty	30.1	9	9	18
**Sub-total residents**		**21**	**26**	**47**

***Educators***				
Family medicine	45.6	7	9	16
Specialty	52.3	11	1	12
**Sub-total educators**		**18**	**10**	**28**

***Program directors***				
Family medicine	56.2	2	2	4
Specialty	47.4	8	1	9
**Sub-total directors**		**10**	**3**	**13**

***Associate deans***	**56.0**	**3**	**1**	**4**

**TOTAL**		**52**	**40**	**92**

### Generalist-specialist collaboration in the formal curriculum: non specific and scattered competency objectives

Content analysis of official documents revealed collaboration competency objectives in all programs. These were institutional, diverse, and mentioned under different competencies.

In describing collaboration skills, specialty programs generally referred to the "interprofessional team" or "other health professionals", without specifically mentioning family physicians. All specialty programs identified referral and consultation skills under the "Medical expert" competency; three referred to specialists' responsibilities in the continuing education of generalists under the "Scholar" competency; two internal medicine and three psychiatry programs mentioned the importance of communicating information about patients to their family physician upon discharge from the hospital under the "Communicator" or "Collaborator" competencies; and four programs had indications such as "Demonstrate the attributes of a good consultant" under the "Professional" competency. All family medicine programs mentioned "learning to make appropriate and timely referrals" under the "Skilled clinician" principle; no other indication concerning relationships with specialists was found.

This lack of detail and uniformity across programs was echoed in our interviews. All residents considered consultation skills a competency to be mastered, but generally indicated that learning intra-professional collaboration was not formalized in clinical rotations, except for psychiatry programs. Collaboration was learned "on the job", and location appeared to play a critical role. Indeed, indications were found that rural settings might favour better generalist-specialist collaboration, compared to university hospitals, where it was not considered a priority.

"I am in a region. Here, as family physicians, we have very close contacts with the basic specialties.... It's a good experience because they [residents] don't often have the opportunity to see this kind of teamwork between family physicians and specialists, with a case management role for the family physician and a consulting role for the specialist." *(Family physician educator)*

"Not so much here [university hospital], but in other places some of the internal medicine services [wards] had a policy whenever they discharged a patient, they would make a courtesy call to their doctor so that they would know that the patient had been in the hospital... but again, that didn't happen every day...." *(Internal medicine resident)*

### Collaboration as experienced in practice in academic medical settings

Even if collaboration between family physicians and specialists is not always formally addressed in the curriculum, the academic training settings provide many occasions to experience it. From the interviews, it is clear that the referral/consultation process sets the stage for collaboration. Generally speaking, respondents expressed similar ideas on what constitutes effective collaboration: clinical relevance of the referral, good communication skills, and clear definition of responsibilities. However, in practice, collaboration runs up against certain obstacles and does not always meet expectations. Many issues were raised such as lack of clarity about the reason for referral or about the results of the consultation and confusion about each others' roles. Lack of time, fee schedule that does not permit reimbursement for telephone consultations and inadequate information systems were also identified as major irritants in day-to-day practice, mainly by the educators. Yet, many comments revealed more deeply-rooted problems. Those problems were classified under two major themes related to Abbott's conceptual framework: issues of professional responsibility and recognition of mutual expertise; and expanding distances between family practice and specialty care in the workplace and in the training programs.

#### Issues of professional responsibility and questioned expertise

Frequently experienced problems were related to inappropriate acknowledgment and coordination of roles. For example, specialists were uneasy leaving it to family physicians to follow their advice:

"Family doctors must realize that, when we do write back to give them advice, we expect that the advice will be heeded. We do appreciate it when we send our advice in the form of consult letters to family doctors and the advice is recorded and recognized." *(Internal medicine program director)*

For vulnerable clienteles, they worried family physicians might not be sufficiently available or have the resources for follow-up:

"I have seen patients that I'd seen in consultations: it didn't work out to send them back to the family doctor. They came back to the hospital because they've been getting gradually sick over a period of two, six, eight weeks, without anybody being able to identify... they were not able to get through to their family physicians." *(Internal medicine educator)*

Family physicians, conversely, were annoyed when specialists "took over" their patients–particularly when they referred them to other specialists–because responsibilities were often unclear:

"I have to refer to specialists who don't know me and who act as if they have control over the patient entirely without any input from me. Specialists being very unclear in their letters.... 'This patient should have *X*, *Y*, and *Z*.' Does that mean you've ordered it? Does that mean I'm supposed to order it? Are you going to follow this patient? Not clear...." *(Family medicine educator)*

It was the specialty residents who had more perspective on the challenges of learning to work as consultants in collaboration with family physicians.

"In my two years as a senior resident, what I learned was how to be a good consultant. It isn't easy. Particularly in internal medicine, where we want to do it all, control everything, while the consulting role is about learning to be clear in our oral and verbal communication, to let people make their own decisions while offering alternatives." (*Internal medicine resident*)

"The other issue with general internal medicine is oftentimes they [family physicians] refer a specific problem to us, so we investigate it further. And in the process of doing the history and physical there's another issue that needs to be dealt with, or some unexplained weight loss... And I find that a little surprising sometimes, to be honest, that a really obvious physical finding might have gone undetected, or the potential implications of it were not identified. And the other thing that I'm a little bit concerned about is whether or not there are slightly different standards as well, depending on where you are." (*Internal medicine resident*)

When questioned about his supervisor's advice regarding such a situation, this resident responded that most often the decision was taken to pursue the investigation without notifying the referring physician.

Overall, we noted that the educators were generally less reflective than the residents about the challenges of the consultation process and expressed more stereotyped negatively tainted experiences of intra-professional collaboration.

#### Expanding distances between specialty and family medicine in the workplace and in the training programs

Queried on the reasons for these collaboration issues, our respondents noted expanding distances between family physicians and specialists regarding the workplace and training settings, two key fields of professional identity enactment and development according to Abbott's theoretical framework.

Many family medicine and specialty educators noted the distance introduced by family physicians' gradual shift from hospitals to private offices and community settings and its impact on collaboration:

"And that's what's been lost by the family doctors leaving the hospital environment.... they used to see their patients in the hospital, used to assist on their own surgeries and everything, they developed relationships with specialists." *(Family medicine educator)*

Collaboration was also said to be neglected as both generalists and specialists are isolated in their respective work settings, due in part to organizational limitations regarding the transfer of information:

"We don't have a good system for communicating what's going on in the hospital to the family doctors. And a good hunk of that is our fault, I don't doubt it, because it's a time-consuming process to track down the family doctor; they're not in the office when you call them, they call you back and you can't remember the specifics. It's a very tedious process and not very many of them come into the hospital anymore to see patients." *(Internal medicine educator)*

"There seems to be little commitment on the part of many of the specialists to facilitating the care provided at the primary care level". *(Family medicine educator)*

Our respondents' discourses also revealed that this distance in the workplace arena–attributable to the evolution of the health care system–was accentuated by another important one resulting from the evolution of the medical training curricula. Indeed, program directors and specialty educators attributed the relative lack of generalist-specialist interactions to the fact that family medicine programs generally moved their residents out of specialized rotations in teaching hospitals and into community-based training:

"They [generalists] ... suggest that their training program should be done in the communities so they disenfranchise their trainees at the beginning and make them different from anybody else ... and therefore they lose the skills that all the other groups have within the hospital scene. ... They learn not to work with the other acute-care or the other specialties and so they're distancing themselves even more." *(Internal medicine educator)*

Consequently, many specialists we interviewed said they were unfamiliar with family physicians' current training. This situation led some of them to believe it was done "at a discount", a situation we found to be associated with the issues of professional responsibility and the questioning of expertise mentioned earlier:

"I wouldn't have a clue as to where [family physicians] are getting trained. There hasn't been a single family practitioner to come through our training program in years.... that's a real problem because I think family physicians do an extraordinary amount of mental healthcare.... I don't know if they're trained for it." *(Psychiatry program director)*

In the view of some educators and many associate deans of postgraduate studies, this second distance is rooted in part in the fact that there are two systems for defining the functions of a physician depending on whether you are talking about a family physician or a specialist. This complicates the integration of residents in formal teaching activities:

"Oh, CanMEDS has seven roles, but Family Medicine has four principles... I know that there is a 'turf' issue here, but I wish the two colleges would get together and call their roles the same thing. You know, the four principles have all of the CanMEDS roles in them. If you just break them down, you can find them. It would be helpful if all our students would have all the same names for their roles. We now have actually adopted the CanMEDS roles as primary initiatives in the undergrad curriculum. It would make family medicine equal to all the other specialties, as opposed to being off by itself." (*Associate dean of postgraduate studies*)

"What would I do differently? I would take away the two-class system of training and make all trainees go through much the same training." (*Internal medicine educator*)

Finally, two specialist respondents alluded to a perceived lack of contribution of family physician educators to the production of scientific medical knowledge–which is another important aspect of the professional system in Abbott's theory–as contributing to the observed problems of collaboration:

"It has been an issue in the training program ... specialists have not viewed family physicians as being at the same level ... because they're not doing the same kind of other academic work that the specialists are doing. I think as soon as we see a family physician who is just as involved in scholarly activity, I think the equal, mutual respect would be the same as two specialists talking to each other." *(Associate dean of postgraduate studies)*

## Discussion

Our findings offer a deeper understanding of current shortcomings in teaching generalist-specialist collaboration. Analysis of the participating programs' objectives, combined with interview data, revealed that mastery of this competency lacked consistency and specificity across programs, with related training objectives scattered under several different competencies. Also, analysis of our respondents' statements brought up deeply-rooted obstacles to effective collaboration that cannot be reduced to issues of consultation or service organization.

Our respondents described distances created between general practice and specialty medicine in the academic settings as a workplace and as a teaching environment. According to Abbott's framework, their discourses indicate that a significant amount of internal differentiation has occurred within the medical profession between family physicians and specialists in the teaching setting. Fragmentation of care in the workplace, due to the evolution of the health care system, has contributed to physicians' concerns and poor understanding of their colleagues' practice conditions. Furthermore, the divergent evolutions of the training programs seem to have also contributed to the divide between family medicine and specialties. While the words "trust" and "distrust" were never used, trust-related issues between family physicians and specialists were raised by all categories of respondents and appeared to be as important as those reported when collaboration between the medical profession and other health professions is considered.[18] These results support the assertion that the differentiation of family physicians' and specialists' professional identities in the teaching setting has an incidence on their collaboration.

Our data also suggest that teaching collaboration between family physicians and specialists is not high on the academic agenda and that the current training strategies and systems may be in part perpetuating the problem. It is interesting to note that, within our sample of respondents, residents were spontaneously more reflective on the question of intra-professional collaboration than were their educators, particularly residents in internal medicine and psychiatry. Among the educators, the specialists were more likely to express concerns about the increasing distance in the training curricula than were the family physicians. However, educators and residents in family medicine seemed bitterer about their overall experience of collaboration.

### Strengths and limitations

The strength of this study is in its analytical approach based on Abbott's systemic theory of professions.[13] Few authors have applied such a robust theoretical framework to the study of collaboration within a profession's sub-disciplines. Also, having respondents of diverse statuses in the training continuum as a whole was invaluable since not everyone was sensitive to the same issues of professional collaboration.

We are confident our observations are credible. Given Canada's national accreditation process and the consistency of themes evoked by our respondents, selected to represent the different regions and missions, we strongly believe our data reliably depicts the situation in Canadian medical academic settings. The recurrence of themes in successive interviews confirmed that saturation was reached and that using two interview methods did not bias our results.

However, there were indications in interviews of generalist-specialist interactions being more effective in rural contexts. Because this question could not be thoroughly explored in this study, we are cautious in stating that our findings may be more applicable to practice conditions in urban academic teaching settings. As to whether the specificities of the Canadian healthcare system affected our observations, we note that many of the tensions reported here have been observed elsewhere, such as in the US[2] and Europe[3]. Our observations might have been accentuated because of the co-existence of two systems of defining competencies in Canada, but references to family medicine's unique character as opposed to specialty medicine can be found in the position statements of most Western family medicine organizations [24-27]. Our analysis may not translate as well to non-Western countries, however, due to differences in training family physicians and specialists, although they may be faced with similar challenges as many are developing their primary care workforce.

## Conclusion

The professional distances noted here between generalists and specialists educators and trainees appear theoretically and empirically significant. They call for a reconsideration of current approaches to teaching intra-professional collaboration. Surely, the teaching of collaboration between specialists and generalist physicians merits as much attention as is currently devoted to inter-professional collaboration in the healthcare sector, and poses comparable challenges. Developing learning activities that foster the mastery of generalist-specialist collaboration while respecting each discipline's needs in terms of training environments remains an educational challenge, as is the case for inter-professional collaboration. Since the study was completed, both Canadian colleges revised their positions, and the CanMEDS roles are now used as the reference to physicians' roles independently of their discipline, although family medicine retained its four overarching principles to describe its specificity. This was decided to facilitate the development of conjunct teaching activities at the residency level and conjunct faculty development activities.

However, many of the obstacles identified are directly related to the evolution of the health care system and the need for hospital settings to be more responsive to the primary care sector. Community hospitals are probably in a better position to adapt and it is not surprising that some of the residents we interviewed reported more positive experiences of collaboration between family physicians and specialists in rural hospitals. We could not explore specifically why this is so in this study, but we can hypothesize that family physicians have closer interactions with their specialist colleagues in the rural community setting as most of them still provide hospital care.

In this regard, many of our respondents proposed bringing generalists back into the hospital as a solution to some of the problems mentioned here. However, like others, we believe this would run counter to the current evolution of health services. Models that bring specialists and generalists closer together in the community, where most care is now provided, would certainly seem more coherent, and academic urban teaching hospitals cannot shy from their responsibility to lead the way if they are to train the physicians of tomorrow.[8,25] We feel the challenges to train future family physicians and specialists to collaborate effectively will not be met only through educational innovations; academic settings should also engage in the development of innovative models of care delivery.

## Competing interests

Since the study was completed, Louise Samson has become President of the Royal College of Physicians and Surgeons of Canada (2006). Marie-Dominique Beaulieu is a member of the Collaborative Action Committee on Intra-Professionalism (July 2007), a joint Committee of the College of Family Physicians of Canada and of the Royal College of Physicians and Surgeons of Canada, whose mandate is to oversee productive action on the recommendations in the CFPC-RCPSC discussion paper entitled "Family Physicians and Other Specialists: Working and Learning Together" (2006).

## Authors' contributions

MDB, LS and LB were involved in every part of the study. GR and MR were involved in the conception and design of the study and in the analysis and interpretation of data. CDG made a substantial contribution to analysis and interpretation of data. MDB and CDG drafted the article. All authors revised the manuscript critically and approved the version to be published.

## Authors' information

MDB is a family physician and is holder of a research chair in family medicine. LS is a radiologist involved in medical education at the Faculty of Medicine of the Universite de Montreal. LB is a social worker and works on interprofessional collaboration. He has been president of the Association of Social Workers of the province of Quebec. GR is a renowned sociologist working on the sociology of reforms in education and healthcare. MR was, at the time of the study, a doctoral candidate in sociology at the Centre de recherche en droit public and is presently employed as a research professional at the Quebec Ombudsman's Office. CDG is a sociologist working as a research professional in family medicine at the Centre hospitalier de l'Universite de Montreal Research Centre (CRCHUM).

## Appendix 1

In this paper, we use "family physician" and "general practitioner" interchangeably to refer to the physician who deals with a variety of problems in a population of patients regardless of age and gender, in contrast with "primary care physician" that includes, in the US, specialties such as pediatrics, obstetrics/gynecology, and internal medicine.

## Pre-publication history

The pre-publication history for this paper can be accessed here:


